# The Influence of Soft Tissue Therapy on Respiratory Efficiency and Chest Mobility of Women Suffering from Breast Cancer

**DOI:** 10.3390/ijerph16245092

**Published:** 2019-12-13

**Authors:** Katarzyna Domaszewska, Tomasz Pieńkowski, Arkadiusz Janiak, Dorota Bukowska, Maria Laurentowska

**Affiliations:** 1 Faculty of Health Sciences, Poznan University of Physical Education, 61-871 Poznań, Poland; laurentowska@awf.poznan.pl; 2 Province Polyclinic Hospital, 62-504 Konin, Poland; pienkowskigryf@o2.pl; 3 Faculty of Rehabilitation and Sport, The President Stanislaw Wojciechowski State University of Applied Sciences in Kalisz, 62-800 Kalisz, Poland; arjan7@wp.pl; 4 Department of Neurobiology, Poznan University of Physical Education, 61-871 Poznań, Poland; bukowska@awf.poznan.pl

**Keywords:** spirometry, VAS Pain, oncology rehabilitation

## Abstract

The aim of the following paper was to determine the influence of soft tissue therapy on respiratory efficiency and chest mobility of women suffering from breast cancer. This study was a controlled, randomized trial. Tests were carried out in a group of patients (*n* = 49) who were hospitalized in the Province Polyclinic Hospital, Konin, Poland. In the study group, irrespective of the standard physical therapy program, an additional therapy program was run. The program consisted of applying specific techniques of soft tissue treatment. All patients in each term were subject to pulmonary function tests, chest mobility, and pain assessment. Statistical analysis of the obtained results of spirometry and chest mobility assessment has revealed no differences in the analyzed parameters between the examined groups in the period of joint therapeutic treatment. In the period between the third examination and the end of the 11-month-rehabilitation treatment, statistically significant differences were observed in the analyzed spirometry parameters; however, there was no difference in the parameters describing airflow in small airways (maximal expiratory flow at 50% (MEF_50_), peak expiratory flow (PEF) between individual groups during consecutive examinations in the course of diversified therapeutic treatment. Chest mobility assessment of the patients, performed during diversified therapeutic treatment, revealed statistically significant differences between the groups. However, there was no difference between the examined groups as far as pain sensation is concerned. Enhancing the regular rehabilitation program by including additional therapeutic methods, which are based on myofascial release and post-isometric relaxation techniques, had beneficial effects regarding respiratory system efficiency.

## 1. Introduction

Breast cancer is the most common type of cancer among women worldwide and the most frequent cause of death. Women suffering from breast cancer constitute 25% of female patients who undergo oncological treatment [1]. Statistics clearly show that this type of cancer mostly affects women over 50 years old [1,2]. Other risk factors include reproductive, mutation in BRCA 1 and BRCA 2 genes, and hormonal factors [3,4,5,6]. Treatment options in the case of breast carcinoma include surgical treatment, systemic treatment (hormonal therapy and chemotherapy), and radiotherapy. Surgical treatment is the basic and indispensable procedure in the case of breast cancer. Early consequences of surgical treatment are connected with immobilization and an extensive postoperative wound. They include respiratory disorders, reduced chest mobility due to postoperative pain, circulatory disorders, for example, orthostatic disorders, as well as reduced muscle strength [7,8]. At a later stage, the deterioration of respiratory efficiency is caused by the impact of ionizing radiation on the lung tissue of female patients undergoing radiotherapy. Ionizing radiation could also cause non-specific inflammatory lesions in the lung tissue, which lead to the formation of atelectasis foci and fibrosis. Histological examination reveals swelling of the interstitial tissue and capillaries, as well as thickened interalveolar-septum [9]. Such changes cause permanent damage to the pulmonary parenchyma and its stiffening, which leads to a significant decrease in respiratory system efficiency. Late consequences of breast cancer treatment include possible neuropathy in the operated area, overgrowth of the postoperative scar, reduced muscle strength in the pectoral girdle and in the upper limb, sensory disturbances, and phantom pain. Additionally, pharmacological treatment may be the cause of reduced efficiency, fatigue, and general weakness. Breast amputation leads to disturbances of the body posture of the operated women, causing the winging of the scapulas, lifting of the shoulders, and curvature in the cervical and thoracic spine [10]. This may result in the asymmetry of the shoulders, scapulas, and waistline [11]. Breast cancer treatment can also have undesirable consequences related to motor organs. Balance disorders of the muscles of the trunk and the changes in statics and body posture of women undergoing treatment lead to reduced chest mobility. This, in turn, may constitute one of the major factors causing reduced functions of the respiratory system by disturbing breathing mechanics. Physiotherapeutic treatment is an integral part of treatment in the case of patients with breast cancer. It enables patients to regain physical fitness and to reduce side effects of the treatment [12,13]. Rehabilitation also constitutes an important factor, which enables patients to be professionally active again [14]. Being able to return to work has a direct impact on enhancing the quality of life of the patients and on faster psycho-physical recovery. Patients who have undergone mastectomy receive standard physiotherapeutic treatment. However, in order to improve their recovery, it is possible to introduce some additional procedures, such as soft tissue techniques, for example, myofascial release and post-isometric relaxation, which help to remove muscle and fascia stiffness in the area of the postoperative scar [15]. Other techniques, which are not commonly used, but which could be applicable to improve the recovery, are thoracic rib and joint mobilization, trigger point therapy, and kinesiotaping [16]. The above-mentioned therapeutic methods can improve the functions of the respiratory system by restoring correct chest mobility and improving the work of respiratory muscles in the operated area. The application of such techniques helps to remove any restrictions or increased muscle tone in the area of the postoperative scar, thoracic fascia, as well as in the myofascial units. It also helps to restore limited fascia mobility, which can improve chest mobility. Soft tissue therapy also improves the activity of respiratory muscles situated in the area of the damaged thoracic fascia, which show contractures and hypertonia, as a result of abnormalities in the fascial canals. The aim of the following paper was to determine the influence of soft tissue therapy on respiratory efficiency and chest mobility of women suffering from breast cancer.

## 2. Materials and Methods

**Figure 1:**
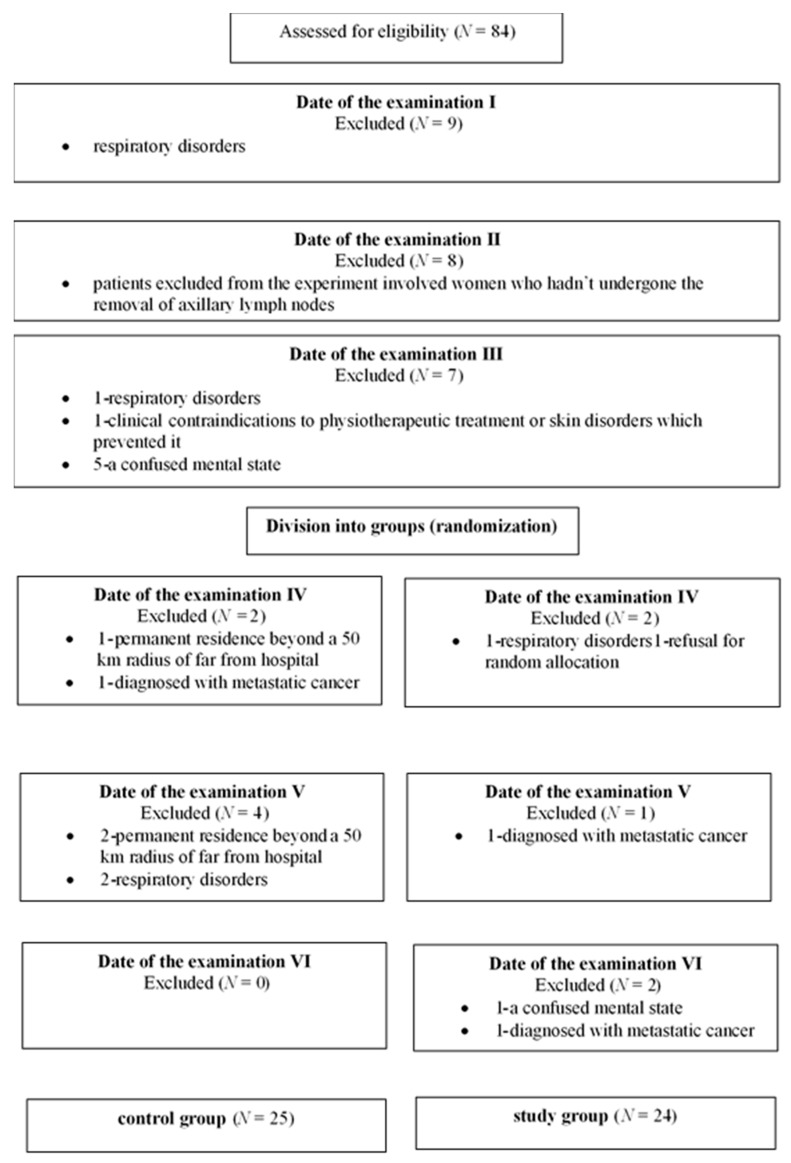
Diagram the course of patients recruitment.

This study was a controlled randomized trial. The study initially enrolled 84 patients that were randomly split into the study and the control group after informed consent was obtained and stratification by the surgical procedure planned at the time of recruitment into the study. The study was conducted according to the Declaration of Helsinki and the National Statement and Human Research Ethics Guidelines and approved by IRB (Institute for Research in Biomedicine) at the Poznan University of Medical Sciences (10 May 2013; Ethics Approval Number: 302/13). An information sheet was provided to each woman approached to participate in the study, and on agreement to participate, informed written consent was obtained. The study was conducted in the Department of Surgical Oncology and in the Department of Rehabilitation over a 12-month period (from 2 June 2013 till 20 July 2014). All patients underwent the oncological treatment shortly after being diagnosed with cancer, and all of them were in the second and third stage of the disease before starting the treatment. The examined women underwent the preoperative procedure (systemic preoperative treatment), operative, and postoperative treatment. The inclusion criteria were the diagnosis of primary breast carcinoma and eligibility for surgical treatment—the modified radical mastectomy of Patey. All patients underwent an operation during the first two months of the project duration. All women participated in the research project for 12 months, regardless of the date of the first spirometry test. Out of 84 women initially enrolled in the project, 35 were excluded from the project at some stage. Excluded from the experiment were women who had not undergone the removal of axillary lymph nodes (8 women), or who were suffering from respiratory disorders, which could affect spirometry results, such as: pulmonary tuberculosis, bronchial asthma or chronic obstructive pulmonary disease, or patients who were taking medication affecting airways patency (13 women); suffered mechanical injury of the chest, for example, after rib fracture or thoracic spine injury, or were diagnosed with metastatic cancer (3 women); had clinical contraindications to physiotherapeutic treatment or skin disorders which prevented it (1 woman), a confused mental state or ability to follow the exercise guidelines for each group (6 women); had permanent residence beyond a 50 km radius from hospital (3 women); refused for random allocation (1 woman) (Figure 1).

Tests were carried out in a group of patients (*n* = 49) who were hospitalized in the Province Polyclinic Hospital, Konin, Poland. Before the surgery, they were administered chemotherapy based on anticyclones and toxoids. The type of treatment scheme and treatment duration depended on the stage of cancer according to the tumor-node-metastasis (TNM) staging system (stage IIB and IIIA). The next stage of treatment was radiotherapy. A dose of 40–42.5 Gy was routinely applied in 25 fractions on the whole breast and in 15–17 fractions at the site of tumours resection. Each of the participants also completed a hormonal treatment with tamoxifen and additionally with a gonadotropin-releasing hormone agonist depending on the stage and the presence of recurrence risk and the patient’s age. A medical examination was conducted 6 times during a year (spirometry, chest mobility, pain assessment). The first examination was carried out on the day when patients were admitted to the Department of Surgery (preoperative examination); the second examination was conducted on the fourth day after the surgery, the third—one month after the operation, the fourth—three months after the surgery, the fifth—six months after the operation, and the sixth—twelve months after the surgery. In the period between the 1st and 3rd examination, patients received the same therapeutic and rehabilitation treatment. This included gradual verticalization, exercises to prevent blood clots, breathing and equipment- free, self- assisted exercises of 10–15 min, 3 times a day, repeated 5–10 times, supervised by a physiotherapist. After the third examination, the patients were split into two groups, each with a different rehabilitation scheme. Over 11 months, both groups (the study group and the control group) performed equipment-free, general-conditioning and breathing, self-assisted exercises of 30 min twice a day, supervised by a physiotherapist in outpatient settings. The study group, apart from the standard procedure, received additional treatment, including soft tissue therapy, such as myofascial release and post-isometric relaxation in the area of the muscle fascia and the postoperative scar. The treatment was performed twice a week by a qualified physiotherapist and involved the relaxation of upper quadrant according to C. Manheim, general chest relaxation on both sides, general relaxation of the operated side of the chest, relaxation of lateral fascia of the trunk, myofascial relaxation of the pectoral major muscle and pectoralis minor, relaxation of the intercostal muscles using ischemic compression, post-isometric relaxation of pectoral muscles, scar treatment (vertical traction, diagonal stretching, static and dynamic rolling) [17]. To minimize potential errors introduced by the treating physiotherapist and medical staff, the women were asked not to inform the physiotherapist and medical staff about participation in the control or study group.

### 2.1. Pulmonary Function Test

The evaluation of pulmonary function was performed by conventional spirometry using a spirometer (Spirobank USB; Medical International Research, Rome, Italy). The directly evaluated parameters were lung volumes, capacities, and flows through the procedures of slow vital capacity (SVC), forced vital capacity (FVC), and maximal voluntary ventilation (MVV), performed in this order at least three times each, according to the standards of the American Thoracic Society (ATS) and the European Respiratory Society (ERS), in the sitting position [18]. Results were expressed as absolute values and as percentages of the reference predicted values from Pereira et al. [19]. By means of the SVC procedure, it was possible to obtain vital capacity (VC). The FVC procedure allowed for the determination of the forced expiratory volume in one second (FEV_1_), FEV_1_/FVC ratio, maximal expiratory flow at 50% (MEF_50_) and peak expiratory flow (PEF). The MVV was expressed in L/min and as percentages of the reference predicted value. Examinations were conducted six times in the course of the project.

### 2.2. Chest Mobility

Chest circumference at maximal voluntary inspiration (Cinsp) and at maximal voluntary expiration (Cexpir) and chest expansion (CE) (the difference between Cinsp and Cexpir) were measured in sitting position using a tape measure marked in 0.1 cm increments at the level of the fourth intercostal space. All women were informed about the examination and were asked to exhale as much as possible and hold the position for Cexpir measurements and to take as deep a breath as possible and to hold it for Cinsp measurements [20]. The highest value of Cinsp and the lowest value of Cexpir in three attempts were recorded for all women, and the difference between Cinsp and Cexpir was recorded as CE. Examinations were conducted six times in the course of the project.

### 2.3. Pain Assessment

The intensity of pain in the operated region of the anterolateral chest wall was measured 6 times in the course of the project, using a 10-degree pain scale (Visual Analogue Scale (VAS) Pain). The VAS Pain Scale is a unidimensional measure of pain intensity, which has been widely used in diverse adult populations [21]. The pain VAS is a continuous scale comprised of a horizontal (HVAS) or vertical (VVAS) line, usually 10 cm (100 mm) in length, anchored by 2 verbal descriptors, one for each extreme symptom [22,23].

### 2.4. Statistical Analyses

The distribution of normality was measured using the Kolmogorov-Smirnov test. A significance test for two mean values, measured in six consecutive examinations, was performed using a non-parametric test of a one-way analysis of variance for repeated measure study ANOVA for related groups. Tukey’s post hoc test was performed to assess the significance of differences between groups, while confidence intervals (CI 95%) were also calculated. The Mann–Whitney U test was employed for non-normally distributed variables, respectively, to evaluate the significance of differences between date of the examination (I and II). The significance level for all statistical analysis was set at *p* ≤ 0.05. All values were presented as mean ±SD. All statistical analyses were performed using STATISTICA 13.0 software (StatSoft, Tulsa, OK, USA).

## 3. Results

Spirometry, chest mobility test, and pain assessment were performed during each examination in the spirometry laboratory of the Province Polyclinic Hospital in Konin by the same people and using the same measuring methods. Standard rehabilitation treatment and soft tissue therapy were performed by one physiotherapist specializing in manual therapy. Ultimately, statistical analysis was conducted, on the basis of the results of 49 patients: 25 from the control group and 24 from the study group with a mean age of 55.33 ± 11.77 years (range 32–77 years). Demographic and other preoperative characteristics are listed in Table 1. Measurements were performed for each of the examined groups six times during the year of project duration.

Both examined groups showed similar anthropometric factors during the whole duration of the project. No changes in body weight were observed between consecutive examinations. Spirometry results are presented as percentages of predicted normal values of the examined parameters during each examination of both analyzed groups. Change of (VC, FEV_1_, FEV_1_/FVC, MVV (Table 2), MEF_50_, PEF (Table 3), chest mobility and pain scale (Table 4).

**Figure 2:**
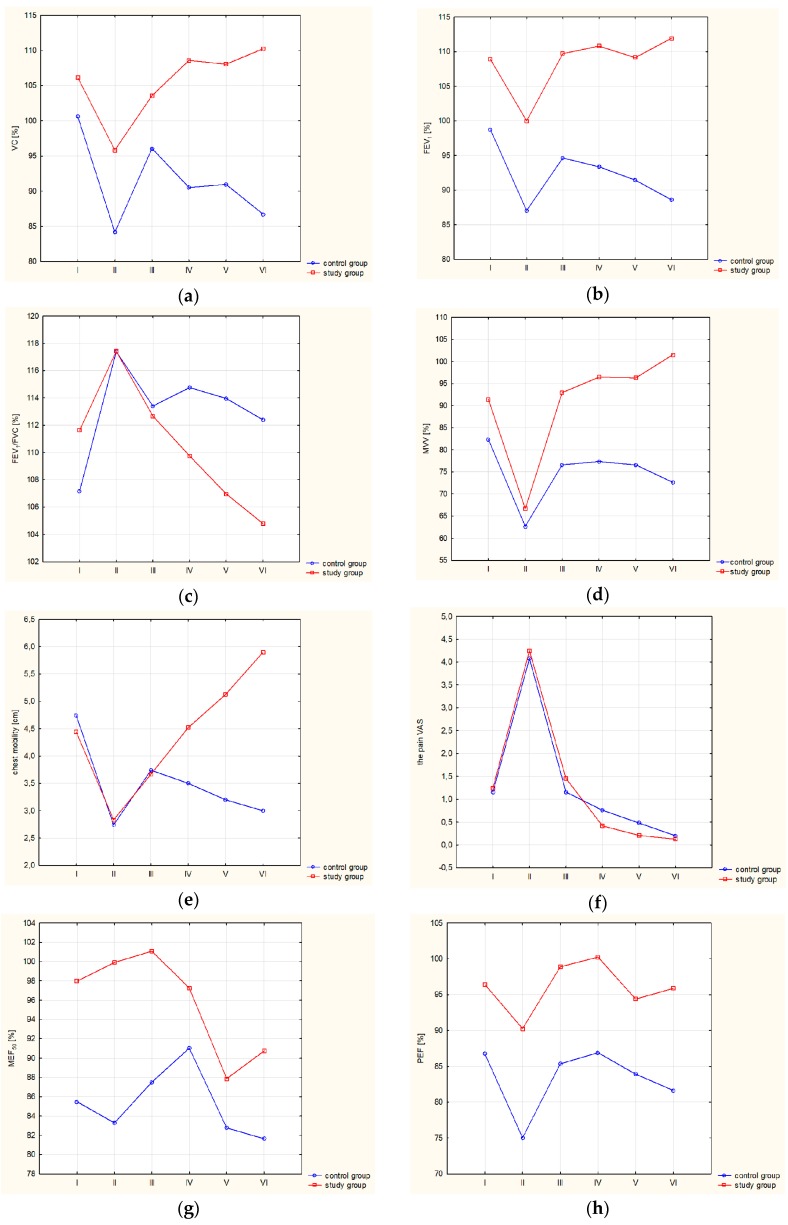
Presentation of the course of changes in the parameters evaluating respiratory efficiency, chest mobility, and pain sensitivity of both groups during the project. (**a**) Changes in vital capacity (VC) (%), (**b**) changes in forced expiratory volume in one second (FEV_1_) (%), (**c**) changes in FEV_1_/forced vital capacity (FVC) (%) (**d**) changes in maximal voluntary ventilation (MVV) (%), (**e**) changes in chest mobility (cm), (**f**) pain scale (VAS Pain), (**g**) changes in MEF (%), (**h**) changes in PEF (%).

Statistical analysis of the obtained results of spirometry and chest mobility assessment has revealed no differences in the analyzed parameters between the examined groups in the period of joint therapeutic treatment. In the period between the third examination and the end of the 11-month-rehabilitation treatment, statistically significant differences were observed in the analyzed spirometry parameters (VC, FEV_1_, FEV_1_/FVC, MVV); however, there was no difference in the parameters describing airflow in small airways (MEF_50_, PEF) between respective groups, during consecutive examinations in the course of diversified therapeutic treatment. Chest mobility assessment of the patients, performed during diversified therapeutic treatment, revealed statistically significant differences between the groups. However, there was no difference between the examined groups, as far as pain sensation is concerned. Figure 2 presents the course of changes in the parameters evaluating respiratory efficiency, chest mobility, and pain sensitivity of both groups during the project.

All the spirometry parameters, as well as chest mobility and individual pain intensity, significantly changed in both groups in the period between preoperative examination and the III examination, which was conducted one month after the I examination (ANOVA *p* < 0.05). The surgery caused a considerable decrease in spirometry parameters during the II examination; VC%, FEV_1_%, FEV_1_/FVC% (Mann–Whitney U-test *p* < 0.01), MVV%, PEF%, chest mobility and individual pain sensitivity (Mann–Whitney U-test *p* < 0.001) for each examined group. Rehabilitation treatment, extended by physiotherapeutic techniques of soft tissue therapy, led to a significant improvement in the analyzed parameters of respiratory system efficiency (ANOVA *p* < 0.05). As far as the control group is concerned, in the period between the III and the VI examination, a statistically significant decrease was observed in VC%, FEV_1_%, FEV_1_/FVC%, and MVV% (ANOVA *p* < 0.05). Both examined groups displayed a significant decrease in PEF and MEF_50_ (ANOVA *p* < 0.05) 11 months after the surgery. In the group that received extended therapeutic treatment, chest mobility improved considerably in the period between the III and the VI examination. Pain sensitivity in both examined groups decreased significantly during the project (ANOVA *p* < 0.001).

## 4. Discussion

The most important outcome of the conducted research is the demonstration of the effectiveness of the soft tissue therapy in terms of improving respiratory efficiency and chest mobility of patients undergoing treatment due to breast cancer. The results clearly indicate significant effectiveness of the soft tissue therapy used in the project in order to relieve early and long-term effects of oncological treatment in terms of respiratory efficiency. Surgical treatment may lead, in most cases, to numerous adverse consequences and complications, which frequently appear in the first few days after the operation. The most common problems include pulmonary complications, circulatory complications, and thromboembolism. Factors conducive to respiratory system complications include pain in the operated area, the application of anaesthetics, limited motor activity of the patient, and impaired cough reflex [24]. Studies conducted by Spyropoulou et al. indicate that radiation therapy to the area of the chest, armpit, and supraclavicular fossa, combined with chemotherapy lead to a decrease in respiratory efficiency after three months of the treatment. Another adverse effect of surgical treatment is the reduction of fascial mobility of the postoperative area, caused by mechanical damage to soft tissue during mastectomy, which causes a skin-tight clothes-sensation in patients, leading to reduced chest mobility [25]. On the basis of research conducted by Fourie, four main regions of limited mobility of the fascia in women after mastectomy were defined, namely, the area of the postoperative scar, the area of the armpit and the arm, anterolateral chest wall on the operated side, and the region of teres major muscle [26]. According to Fourie and Kärki et al. limited mobility of the fascia and stiffness in that region could last even up to ten years after mastectomy [26,27]. Having analyzed the effectiveness of soft tissue therapy, the authors decided to apply this rehabilitation model to patients who had undergone breast cancer surgery. Box et al. and Stecco et al. claim that the stiffness of the postoperative scar and limited tissue mobility in the above-mentioned regions can lead to a shoulder joint disfunction, limited mobility of the glenohumeral joint and of the chest [28,29]. The consequence is reduced respiratory muscle efficiency and respiratory muscle fatigability. It is the major cause of limited motor activity of the patients, reduced chest mobility, cough avoidance, and a decrease in the amplitude of diaphragm movement. Postoperative changes described by Pinto et al. and Spyropoulou et al. constitute an explanation for a decrease in spirometry factors in our research, in both analyzed groups during the postoperative period. The reported decrease in respiratory efficiency of both examined groups in the early stage after the surgery was caused by painfulness of the scar and of the area of drain insertion, and by limited motor activity after the operation. The authors emphasize diagnostic importance of the measurement of the following parameters: FVC, FEV_1_, MVV, and of the Tiffeneau test in diagnosing and monitoring the condition of patients who undergo oncological treatment on every stage of the treatment and rehabilitation [24,30]. The application of an additional therapeutic program involving soft tissue therapy undoubtedly affected the improvement in the efficiency of auxiliary respiratory muscles of the patients. The group that received an additional therapeutic program displayed significantly higher values of the VC, FEV_1_, FEV_1_/ FVC, MVV, and chest mobility, especially 6 and 12 months after the operation, which undoubtedly affects the quality of life of the patients. Our studies have shown a considerable decrease in pain intensity of both examined groups in the course of the project (ANOVA *p* < 0.001); therefore, it was not a factor that could significantly affect chest mobility changes or respiratory efficiency in the consecutive examinations. Chest mobility increased significantly in the study group that received additional therapeutic treatment between the III and the VI examination (ANOVA *p* < 0.001). Gradual limitation of chest mobility among patients from the control group was undoubtedly caused by a growing area of tissue restriction in the region of the scar and the frontal chest wall, which was caused by postoperative mechanical damage and radiation-induced damage [31]. Engel and Vemulpad, in their randomized studies on healthy people, showed a beneficial influence of manual soft tissue therapy on FEV_1_ and FVC, in comparison with the effectiveness of physical exercises [32]. The effectiveness of such therapy was also described in the studies conducted by Yimaz from 2016. He described the effectiveness of a single application of manual soft tissue therapy on respiratory muscle strength of patients suffering from chronic obstructive pulmonary disease (COPD). An increase in relaxation and the relaxation of breathing muscles resulted in an increase in chest mobility and a reduction of dyspnea [33]. Published medical literature contains scarce information on the application and effectiveness of soft tissue therapy in the process of the rehabilitation of patients after mastectomy. Hence, the presented results could contribute to formulating new and effective methods of rehabilitation for women who have undergone breast cancer surgery.

## 5. Conclusions

Enhancing the regular rehabilitation program to include additional therapeutic methods, which are based on myofascial release and post-isometric relaxation techniques, had beneficial effects regarding respiratory system efficiency of women undergoing oncological treatment due to breast cancer.

## Figures and Tables

**Table 1:** Selected anthropometric factors during each examination of both analyzed groups.

	Control Group *n* = 25	Study Group *n* = 24
Age (years)	54.52 ± 11.84 (49.63–59.41)	56.17 ± 11.88 (46.15–56.19)
Height (cm)	161.16 ± 6.0 (158.68–163.64)	158.17 ± 6.88 (155.26–161.08)
Weight (kg) I	72.12 ± 10.91 (67.62–76.62)	67.75 ± 12.27 (62.57–72.93)
II	72.12 ± 10.91 (67.62–76.62)	67.75 ± 12.27 (62.57–72.93)
III	71.96 ± 11.00 (67.42–76.50)	67.58 ± 12.13 (62.46–72.70)
IV	72.16 ± 10.59 (67.79–76.53)	67.96 ± 12.57 (62.65–73.27)
V	72.88 ± 10.24 (68.65–77.11)	69.04 ± 12.96 (63.57–74.51)
VI	73.52 ± 10.45 (69.21–77.83)	69.46 ± 13.13 (63.91–75.01)

Values are mean ± SD (confidence intervals 95%).

**Table 2:** Spirometry parameters during each examination of both analyzed groups.

Parameter	Date of the Examination	Control Group = 25	Study Group = 24	*p* Value
VC (%)	I	100.60 ± 16.13 (93.94–107.26)	106.13 ± 16.19 (99.29–112.97)	>0.05
	II	84.16 ± 17.33 (77.01–91.31)	95.83 ± 20.55 (7.15–104.51)	<0.05
	III	96.08 ± 18.66 (88.38–103.78)	103.58 ± 15.97 (96.84–110.32)	>0.05
	IV	90.52 ± 21.12 (81.80–99.24)	108.58 ± 16.35 (101.67–115.49)	<0.01
	V	90.96 ± 22.90 (81.51–100.41)	108.04 ± 18.34 (100.29–115.75)	<0.01
	VI	86.72 ± 17.46 (79.51–93.93)	110.25 ± 18.03 (102.64–117.86)	<0.001
FEV_1_ (%)	I	98.72 ± 22.94 (89.25–108.19)	108.88 ± 18.16 (101.21–116.55)	>0.05
	II	87.04 ± 22.16 (77.89–96.19)	100.01 ± 16.79 (92.91–107.09)	<0.05
	III	94.64 ± 19.82 (86.46–102.82)	109.71 ± 18.32 (101.97–117.45)	<0.01
	IV	93.36 ± 19.11 (85.47–101.82)	110.79 ± 15.61 (104.20–117.45)	<0.01
	V	91.44 ± 21.65 (82.50–100.38)	109.13 ± 16.35 (102.22–116.04)	<0.01
	VI	88.60 ± 17.09 (81.55–95.65)	111.92 ± 17.17 (104.67–119.17)	<0.001
FEV_1_/FVC (%)	I	107.16 ± 21.54 (8.27–116.05)	111.63 ± 9.12 (107.78–115.48)	>0.05
	II	117.40 ± 7.37 (114.36–120.44)	117.42 ± 9.74 (113.31–121.53)	>0.05
	III	113.40 ± 7.29 (110.39–116.41)	112.67 ± 7.46 (109.52–115.82)	>0.05
	IV	114.76 ± 9.61 (110.79–118.73)	109.75 ± 7.07 (106.76–112.74)	<0.05
	V	113.96 ± 7.78 (110.75–117.17)	106.96 ± 6.89 (104.05–109.87)	<0.01
	VI	112.40 ± 8.16 (109.03–115.77)	104.79 ± 8.93 (101.02–108.56)	<0.01
MVV (%)	I	82.36 ± 24.63 (72.19–92.53)	91.42 ± 24.02 (81.28–101.56)	>0.05
	II	62.68 ± 23.49 (52.98–72.38)	66.67 ± 26.38 (55.53–77.81)	>0.05
	III	76.60 ± 22.76 (67.20–86.00)	92.96 ± 27.99 (81.14–104.78)	<0.05
	IV	77.36 ± 22.15 (68.22–86.50)	96.46 ± 20.12 (87.96–104.78)	<0.01
	V	76.56 ± 24.83 (66.31–86,81)	96.29 ± 24.68 (85.87–106.71)	<0.01
	VI	72.64 ± 20.75 (64.07–81.21)	101.54 ± 21.57 (92.43–110.65)	<0.001

Values are mean ± SD (confidence intervals 95%). VC: vital capacity, FEV_1_: forced expiratory volume in one second, FVC: forced vital capacity, MVV: maximal voluntary ventilation.

**Table 3:** Values of the analyzed parameters allowing to assess the patency of small bronchi of both analyzed groups.

Parameter	Date of the Examination	Control Group	Study Group	*p* Value
MEF_50_ (%)	I	85.48 ± 27.68 (74.05–96.91)	97.96 ± 23.30 (88.12–107.80)	>0.05
	II	83.28 ± 25.55 (74.73–95.83)	99.92 ± 34.89 (85.18–114.66)	>0.05
	III	87.52 ± 26.09 (76.75–98.29)	101.08 ± 27.03 (89.66–112.50)	>0.05
	IV	91.04 ± 28.73 (79.18–102.90)	97.25 ± 22.87 (87.69–107.01)	>0.05
	V	82.76 ± 25.54 (72.22–93.30)	87.88 ± 20.45 (79.24–96.52)	>0.05
	VI	81.64 ± 25.11 (71.27–92.01)	90.75 ± 19.83 (82.38–99.12)	>0.05
PEF (%)	I	86.76 ± 19.91 (78.54–94.98)	96.42 ± 21.10 (87.51–105.33)	>0.05
	II	75.04 ± 19.10 (67.16–82.92)	90.25 ± 26.12 (79.22–101.28)	<0.05
	III	85.36 ± 19.25 (77.41–93.31)	98.88 ± 25.43 (88.14–109.62)	<0.05
	IV	86.92 ± 19.05 (79.06–94.78)	100.25 ± 27.49 (88.64–111.86)	≥0.05
	V	83.92 ± 17.89 (76.54–91.30)	94.38 ± 23.61 (84.41–104.35)	>0.05
	VI	81.64 ± 16.48 (74.84–88.44)	95.88 ± 21.81 (86.67–105.09)	<0.05

Values are mean ± SD (confidence intervals 95%). MEF_50_: maximal expiratory flow at 50%, PEF: peak expiratory flow.

**Table 4:** Values of chest mobility parameters and the intensity of pain of both analyzed groups during each examination.

Parameter	Date of the Examination	Control Group	Study Group	*p* Value
Chest mobility assessment	I	4.74 ± 1.22 (4.24–5.24)	4.44 ± 0.73 (4.13–4.75)	>0.05
	II	2.74 ± 0.74 (2.43–30.05)	2.83 ± 0.67 (2.55–3.11)	>0.05
	III	3.74 ± 1.25 (3.22–4.26)	3.67 ± 0.79 (3.44–4.00)	>0.05
	IV	3.50 ± 1.22 (3.00–4.00)	4.52 ± 0.96 (4.11–4.93)	<0.01
	V	3.20 ± 1.08 (2.75–3.65)	5.13 ± 1.02 (4.70–5.56)	<0.001
	VI	3.00 ± 1.09 (2.55–3.45)	5.90 ± 0.92 (5.51–6.29)	<0.001
Pain scale (VAS Pain)	I	1.16 ± 2.70 (0.05–2.27)	1.25 ± 2.42 (0.23–2.27)	>0.05
	II	4.08 ± 2.43 (3.08–5.08)	4.25 ± 1.78 (3.50–5.00)	>0.05
	III	1.16 ± 0.99 (0.75–1.57)	1.46 ± 1.35 (0.89–2.03)	>0.05
	IV	0.76 ± 1.16 (0.72–0.80)	0.42 ± 0.78 (0.09–0.75)	>0.05
	V	0.48 ± 1.05 (0.05–0.91)	0.21 ± 0.51 (−0.01–0.43)	>0.05
	VI	0.20 ± 0.70 (−0.09–0.49)	0.13 ± 0.45 (−0.06–0.32)	>0.05

Values are mean ± SD (confidence intervals 95%).
